# Job-Related and Nonjob-Related Gossips Among Low-Ranked Employees in Unionized Service Organization

**DOI:** 10.3389/fpsyg.2020.00994

**Published:** 2020-06-11

**Authors:** Mohsin Bashir, Rizwan Shabbir, Sharjeel Saleem, Muhammad Abrar, Shahnawaz Saqib, Shahzad Habib Gill

**Affiliations:** ^1^Lyallpur Business School, Government College University Faisalabad, Faisalabad, Pakistan; ^2^Rahim Yar Khan Division, MEPCO, Rahim Yar Khan, Pakistan

**Keywords:** incivility, union members, gossips, low-ranked employees, cynicism, psychological contract violation

## Abstract

Workplace incivility is a common phenomenon that is frequently found across all organizations and cultures. This study was planned to investigate the impact of workplace incivility on job and non-job related gossips through the mediating role of cynicism and psychological contract violation. The perspective of low-ranked unionized employees was explored through a survey method by using stratified sampling in eight strata, which were formulated based on geographical distribution. A total of four hundred questionnaires were distributed among the employees of eight circles, 50 from each, while use able responses remained 301. SmartPLS was used to analyze the data through structural equation modeling. From a theoretical perspective, this study has made several contributions by investigating the impact of workplace incivility in the South Asian context and documenting the impact of incivility from the perspective of individuals belonging to minority socio-cultural status. Besides supporting existing literature, this study provided a unique argument that low-ranked employees in South Asian societies do not spread nonjob-related gossips. This finding is contradictory to the existing literature; and, thus, calls for future research to identify this inconsistency. Limitations and future directions are also discussed.

## Introduction

Workplace incivility acts as a global paradox that exists in business organizations, especially with a diverse cultural background (Cortina et al., 2001; Schilpzand et al., 2014). Among different types of deviant behaviors, workplace incivility (Blau and Andersson, 2005) is the most hazardous for individuals/organizations. Williams and Anderson (1991) defined incivility as “the low intensity deviant behavior with ambiguous intent to harm the target, in violation of workplace norms of mutual respect.” Due to such low-intensity deviant behaviors, organizations bear direct and indirect costs in millions of dollars (Porath and Pearson, 2013). Extant literature has documented toxic impacts of incivility on organization, group, and individual-level outcomes (Schilpzand et al., 2014). Individuals experiencing incivility tend to show less citizenship behavior (Dalal, 2005), higher employees turnover (Chiaburu and Harrison, 2008), high level of stress (Bowling and Beehr, 2006), lower level of engagement (Giumetti et al., 2013), lower job satisfaction (Miner-Rubino and Reed, 2010), marital dissatisfaction that cause work–family conflict (Ferguson, 2012), etc. Terlicki (2011) identified several individual and work characteristics as antecedents of workplace incivility. Lack of communication skills and diminished intellectual capital might have paved the way for the ascension of incivility at the workplace, and experience of incivility might lead to feelings of hostility, aggression, violence, depression, and other workplace and societal outcomes (Akella and Lewis, 2019).

Previous research has investigated the consequences of incivility from affective, attitudinal, cognitive, and behavioral perspectives of the victim (Schilpzand et al., 2014). Majority of these findings are based on the studies that have been conducted in Western and developed countries, such as United States (Viotti et al., 2018), Australia (Griffin, 2010), China (Chen et al., 2013), New Zealand (Griffin, 2010), Canada (Leiter et al., 2011), Singapore (Lim et al., 2018), and United Kingdom (Totterdell et al., 2012). The growing interest of scholars in the incivility phenomenon shows that it has become a global issue (Schilpzand et al., 2014); however, the South Asian perspective has been ignored at large by researchers (Ghosh, 2017). A few studies have been conducted on workplace incivility employing the Asian samples (e.g., Handoyo et al., 2018; Loh et al., 2019). Our study, however, greatly varies from the previous studies, as these have been conducted in Australia, Singapore, and Indonesia. Loh et al. (2019) assessed the impact of workplace incivility on emotional exhaustion, job satisfaction, and work withdrawal using a sample of Australian and Singaporean employees working in various organizations. Handoyo et al. (2018) developed and validated workplace incivility scale using a sample from Indonesia. Moreover, the above-mentioned studies dealt with different variables and were placed in a different context. Our study, on the other hand, is conducted in Pakistan, a Southeast Asian country. Thus, we make an important contribution by placing our study in Southeast Asian context.

Therefore, owing to several reasons, this study has attempted to investigate the largely ignored incivility phenomenon and its consequences from a South Asian perspective. First, South Asian societies are characterized by high-power distance (Hofstede, 1983), and power abuse can foster incivility because high-power individuals believe that they are exempted from the moral rules (Olekalns et al., 2014). Second, incivility is more frequently experienced by the low-ranked individuals (Cortina et al., 2001), and relationship orientation of Asian societies, which stems from identity-based interaction and personalization, can increase the occurrence of incivility (Kakar and Kakar, 2007; Agarwal and Gupta, 2018). These identity-based and personalized interactions, kinship, caste, social class, and religion might lead the lower-level employees to suffer from negative outcomes (Ghosh, 2017).

Third, uncivil behavior in the Western countries may not be considered uncivil in Asia (Ghosh, 2017), as dissimilarities in social and cultural orientation may have an impact on the perception of workplace incivility, and it can be culture specific (Lim and Lee, 2011). Therefore, in Asian societies, low-ranked employees might face severe discrimination at the workplace (LasisiOlukayode et al., 2014) due to their minority sociocultural status. Hence, investigating the phenomenon of workplace incivility and its negative outcomes in individuals of minority sociocultural status might be fruitful. Current studies call for examining the role of hierarchies in shaping high- and low-rank service employees toward mistreatment in a cultural setting (Moon et al., 2018). Although existing body of knowledge pertaining to incivility has addressed various cognitive, attitudinal, and behavior outcomes (Schilpzand et al., 2014), its relationship with job- and nonjob-related gossips has not been explored yet. Based on the above arguments, this study investigated the response of lower-level employees toward workplace incivility regarding job-/nonjob-related gossips through mediating mechanism of psychological contract violation (PCV) and cynicism in unionized public service organization of a patriarchal culture. Lastly, based on the recommendations of Schilpzand et al. (2014), we have selected a unionized public sector organization that is providing utility services in a high-power distance nation. Although the extant research on incivility represents respondents from diverse professions and industries (Schilpzand et al., 2014) across the globe, perceptions of low-rank unionized employees have not been explored yet.

## Theory and Hypotheses Development

Several theoretical frameworks provide support to strengthen our arguments based on the social exchange theory, affective events theory, work environment hypothesis, and job demands–resources (JD-R) model. First, this study is consistent with social exchange theory (Blau, 1964) as “*reciprocity exists when one individual reacts to others*.” This reciprocity is based on an exchange of benefits that are socioeconomic in nature; thus, lower-level employees experiencing incivility could indulge in gossips either job- or nonjob-related gossips as negative reciprocity. Second, affective events theory (Weiss and Cropanzano, 1996) provided a theoretical lens that clarified the relationship between workplace incivility, gossips, cynicism, and PCV. Therefore, events of mistreatment experienced by lower-level employees could trigger negative emotions, and individuals might indulge in gossips (Lim et al., 2008).

Third, the work environment hypothesis (Leymann and Gustafsson, 1996) explains the underlying phenomenon of workplace incivility in Asian societies. Characteristics of perpetrator and targets are not the underlying cause behind workplace incivility; rather, it is an outcome of prevailing environmental conditions within organizations such as high-power distance, patriarchal culture, or socioeconomic status, especially in the case of lower-level employees (e.g., gender, caste, religion, and regional origin). Lastly, the JD-R model (Bakker and Demerouti, 2007) provides a theoretical base to explain the reciprocal link between workplace incivility and gossips. In Asian societies, lower-level employees might involve in gossips when they deplete their emotional resources while coping with mistreatment. According to Leiter et al. (2011), negative events reduce individual’s resources, which can lead lower-level employees to involve in gossips. Thus, high job demands and fewer resources brought exhaustion among employees by pushing them in a situation to discuss negative aspects (Schaufeli and Bakker, 2004).

### Workplace Incivility, Cynicism, and Gossips

Workplace incivility is “*low-intensity interpersonal mistreatment enacted with ambiguous intent to harm the target*” (Andersson and Pearson, 1999) through being rude, discourteous, impolite, or violating workplace norms of behavior. These rude and discourteous behaviors could generate cynical individuals, strained relationships, and an unpleasant work environment. Individuals who face hostile and unethical behaviors at work are likely to develop negative emotional reactions (Weiss and Cropanzano, 1996), leading to harmful consequences by impairing positive attitudes and behaviors at work.

Previous studies have documented the negative consequences of incivility on employee attitudes and behaviors in the shape of low organizational commitment (Lim and Teo, 2009), less satisfaction with job (Miner-Rubino and Reed, 2010), counterproductive work behaviors (Penney and Spector, 2005), decreased work engagement (Chen et al., 2013), higher level of absenteeism (Sliter et al., 2012), and impaired citizenship behavior (Taylor et al., 2012). Incivility is more frequently directed downward (Cortina et al., 2001), and incremental trends can be observed in high-power distance culture where chances of incivility increase due to power gaps (Galinsky et al., 2008). Undesirable behavior is a consequence of misuse of power because employees who enjoy power perceive themselves to be above the rules and obligations (Bowles and Gelfand, 2010). Incivility can adopt various shapes such as discussing other employees in unprofessional manners, using insulting comments, and arrogant tone (Lim et al., 2018). Thus, workplace incivility undermines the dignity, lordliness, and self-esteem of individuals at the workplace (Marchiondo et al., 2017). The reaction to incivility can be immediate, and low-rank employees are likely to develop feelings of anger due to less power (Lim et al., 2018). This state of affairs will drive them to develop negative perceptions regarding the employer, i.e., cynicism. Cynicism is defined as “*a negative attitude toward one’s employing organization, comprising three dimensions: (1) a belief that the organization lacks integrity; (2) negative affect toward the organization; and (3) tendencies to disparaging and critical behaviors toward the organization that are consistent with these beliefs and affect*” (Dean et al., 1998, p5). It occurs when individuals believe that their employer has betrayed them and did not show the integrity and honesty they were expecting (Abraham, 2000; Bedeian, 2007; Yasin and Khalid, 2015). Poor work dynamics, particularly the unachievable prospects of the workplace, gives rise to cynicism (Pate et al., 2000), which is further connected to employee disappointment and hatred toward workplace, management/administrators, and/or other objects in the organization (Andersson and Pearson, 1999). The framework of social information processing theory provides support to this argument. The employees tend to develop attitudes on the basis of self-perceptions based on past events (Salancik and Pfeffer, 1978). Low-rank employees are likely to shape cynicism on the basis of past experiences of incivility. Hence, we hypothesize that:

H1: Workplace incivility is positively associated with cynicism among lower-level employees.

Social information theory (Salancik and Pfeffer, 1978) provides enough aid in understanding the relationship between incivility, cynicism, and gossips. According to this theory, social settings have significant effects on individual attitudes, behaviors, and desires. Being adaptive organisms, employees adapt attitudes, behaviors, and beliefs to their social context and the reality of their own past and present experiences (Kuo et al., 2015). Hence, social standards, environmental aspects, and relationships with others impact any person’s opinions, attitudes, and behaviors. Thus, engaging in gossips might provide a way to lower-level employees for releasing their anger generated in response to incivility. Drawing on the social exchange theory (Blau, 1964), it is contended that individuals indulge in deviant behaviors (Bennett and Robinson, 2003) when they experience incivility at the workplace. In case, when targets of incivility are inferior in organizational hierarchy, reacting with deviant behavior might result in interpersonal conflict (Aquino et al., 2001) and costly to bear, so individuals will opt to follow gossiping behavior as a punishment tool (Decoster et al., 2013). Gossip is “*the practice of producing, hearing or participating in evaluative comments about someone*” (Foster, 2004). At the workplace, gossip is usually seen as informative or entertaining (Ferreira, 2014), but this fun and enjoyment cannot be free of evil. It could hamper peace and organizational justice due to its destructive nature and negativity.

Asian societies are collectivist in nature (Hofstede, 1983), and friendly relationships in social circles can provide room for the arousal of gossips (Kuo et al., 2015). The collectivist nature of Asian societies also ensures familiarity and harmony among groups where people sharing common frame of reference and members are aware of each other’s values and ethics; this might increase chances of gossip (Kurland and Pelled, 2000), and group setting provides sound ground for gossip as it fulfills the human need of belonging (Ben-Ze’ev, 1994). In social circles, gossiper has assurance that his privacy is protected, and he cannot be easily held accountable; this also increases the room for gossip (Rosnow and Georgoudi, 1985). For this study, gossip has been considered from two perspectives: job-related gossip (related to tasks) and nonjob-related gossip, which contains issues pertaining to social or personal life (Kuo et al., 2015). On the basis of the above arguments, it is hypothesized that:

H2a: Individuals tend to involve in job-related gossips in response to workplace incivility.

H2b: Individuals tend to involve in nonjob-related gossips in response to workplace incivility.

H2c: The relationship between workplace incivility and job-related gossips is mediated by cynicism.

H2d: The relationship between workplace incivility and nonjob-related gossips is mediated by cynicism.

### Workplace Incivility, Psychological Contract Violation, and Gossips

The psychological contract is “*individual beliefs, shaped by the organization, regarding terms of an exchange between individuals and their organization*” (Rousseau and Tijoriwala, 1998). It refers to the items and principles in a reciprocal exchange agreement among employees and the organization (Robinson and Wolfe Morrison, 2000; Johnson and O’Leary-Kelly, 2003; Tomprou et al., 2012). This unwritten contract is breached when employees perceived a discrepancy between what he/she has was promised and what is fulfilled (Agarwal and Bhargava, 2013). This perception of breach prompts negative emotions about unmet expectations connected with particular promises (Coyle-Shapiro and Conway, 2005), leading toward generation of negative attitudes (Aykan, 2014). Previous studies have documented its relationship with job satisfaction (Agarwal and Bhargava, 2013), lowered work engagement (Parzefall and Hakanen, 2010), and employee turnover (Ballou, 2013). When such contract is violated, employees feel frustrated and disappointed, and they take out their negative emotions and feelings about their organizations (Kuo et al., 2015). According to Conway and Briner (2005), breaches could be caused by poor work environment (Leymann and Gustafsson, 1996). Poor human resource (HR) policies and lack of managerial support being a component of work environment can provide room for downward mistreatment due to power gaps between low-rank employees and their supervisors (Tepper, 2000). This mistreatment is directly related to deviant behaviors in the organization (Mitchell and Ambrose, 2007; Xu et al., 2012) and puts a strong negative effect on the feelings, emotions, well-being, attitude, and behavior of employees (Zellars et al., 2002). From an organizational context, individuals are inclined to confront mistreatments by low-rank perpetrators, but they avoid confronting high-rank offenders (Porath et al., 2008). This increases the possibility of engaging in gossips due to high potential cost of confrontation against high-rank individuals (Decoster et al., 2013). In social networks, it is difficult to control gossips due to its universal nature. Almost 14% workplace coffee-break chats are gossips, and ∼66% of general talks among employees are related to coworkers (Cole and Dalton, 2009, cited in Kuo et al., 2015). Negative gossiping can be more dangerous to the organization, as it can create hostile environment not only for the people who are being gossiped about but also for those who listen to that gossip (Grosser et al., 2012). Gossip results in employee embarrassment and awkwardness because gossip usually carries private and sensitive topics (Foster, 2004), and mostly, it harms other’s reputation and integrity (Cole and Dalton, 2009). Negative gossips are like a toxin in an organization (Yang et al., 2014). Gossips are uncontrollable, and this phenomenon cannot be eliminated because of its ancient embedded human nature from any context. From the above arguments, it can be assumed that indulging in gossips will be common response when employees experience incivility in high-power distance and patriarchal culture. Thus, we formulate our next hypothesis as follows:

H3a: Workplace incivility predicts PCV among low-rank employees.

H3b: The relationship between workplace incivility and job-related gossips is mediated through PCV.

H3c: The relationship between workplace incivility and nonjob-related a gossip is mediated through PCV.

## Materials and Methods

### Participants

The sample consisted of lower-level employees who are working in a Power Distribution and Maintenance Company in Pakistan named as Multan Electric Power Company (MEPCO). First, lower-level employees were selected as target respondents due to their frequent exposure of incivility within public sector organizations (LasisiOlukayode et al., 2014). This study was conducted in non-Western settings, and due to relational orientation of Asian societies (Kakar and Kakar, 2007), kinship, caste, class, and religion might influence victims of workplace incivility (Ghosh, 2017). Low-rank employees might experience incivility at the workplace due to dissimilarities in social and cultural orientation (Lim and Lee, 2011). Therefore, due to higher power distance (Hofstede, 1983), lower-level service employees believe that their voice/say cannot reform organizational process, and thus, they become pioneer in experiencing a breach (Morrison and Robinson, 1997). Moreover, such service-orientated employees are less inclined toward connecting themselves with the top management, which resulted in a breach of contract (Lester et al., 2002).

### Procedure

This study applied probability sampling technique (Bryman and Bell, 2015), and under the umbrella of probability sampling, stratified sampling was used. In Pakistan, The Water and Power Development Authority (WAPDA) is the sole authority for electricity generation and distribution and one of the largest employers of human resources in Pakistan. A total of 10 distribution companies are working under WAPDA to provide services across Pakistan, and MEPCO is the largest power distribution company with a working strength of 24,854 employees of various cadres, serving across the 13 districts and a population of 33.3 million approximately. Eight strata were framed on the basis of the entire geographical distribution of employees/circles for data collection. Fifty respondents were approached from each circle to constitute a sample of 400 (Krejcie and Morgan, 1970).

Initially, 400 questionnaires were distributed among respondents, keeping in view the general of thumb, i.e., 5–10 questions against each item/statement of questionnaire. A total of 34 items were used in the questionnaire; hence, a sample size of 350 was sufficient for inference purposes; however, a slightly higher sample size was selected. Out of the 400 distributed questionnaires, 335 were received back. Partially filled and incomplete questionnaires were discarded, and at the end, a useable sample of 301 responses was retained for final data analysis. Pilot testing was carried out for 10% of the sample size, i.e., 40 respondents. Reliability values were within the acceptable range, i.e., >0.60. Due to self-reported responses, common method bias (CMB) was likely to prejudice the results, but using self-reported and single-source measures in management research is common (Ng and Feldman, 2013). We, however, employed several measures to minimize CMB. First, we assured respondents regarding confidentiality of their responses; moreover, to avoid monotonic response, some items were reverse coded (Malhotra et al., 2006). The items regarding independent, mediating, and dependent variables were randomly placed in the designed questionnaire supported by research model (Papa et al., 2018). This ensured that respondents could not easily combine related items or identify their correlation, which is required for attenuating CMB (Chang et al., 2010).

### Measures

Five-point Likert scale ranging from 1 (strongly disagree) to 5 (strongly agree) was used. Workplace incivility was assessed through an eight-item questionnaire developed by Cortina et al. (2001) on a scale of every day (5), several times a week (4), about once a week (3), once or twice in a month (2), and once or twice in a year (1). Sample items include “In your organization someone put you down or was arrogant to you in some way,” and “In your organization someone made demeaning, rude, or derogatory remarks about you.” The mediating variable cynicism was assessed by a 12-item questionnaire developed by Brandes et al. (1999), recently used by Bellini et al. (2015) on a 5-point Likert scale. Sample items include “I believe that my organization says one thing does another,” and “when I think about my organization, I feel a sense of anxiety.” The second mediating variable PCV was assessed on the basis of the four-item scale developed by Robinson and Wolfe Morrison (2000). The sample item includes “I feel betrayed by my organization.” The two dependent variables, job- and nonjob-related gossips, were measured on the basis of scale developed by Kuo et al. (2015) having five items for each. The original 20-item version developed by Kuo et al. (2015) covers positive and negative aspects of job- and nonjob-related gossips; however, this study considered only the negative side pertaining to job- and nonjob-related gossips.

### Demographic Profile

Respondents were also asked to report their demographic characteristics (Table 1). First of all, the gender of the respondents was asked from the respondents, and they reported gender status as “male” or “female.” The transgender option was not considered in this study due to the minute portion of the workforce. Individuals reported their employment status as “permanent” or “temporary.” In addition to this, age in years and length of experience were also asked from the respondents. These demographic characteristics were considered as control variables keeping in view the previous studies that show that the psychological contract of temporary staff (transactional) is quite different from that of the permanent ones (relational contract). Similarly, job experience was also considered as control because newly inducted employees can experience incivility up to a great extent in comparison to the older workers (Laschinger, 2012).

**TABLE 1:** Sample description.

**Description**	**Frequency**	**Percent**
**Gender**		
Male	288	95%
Female	13	5%
**Nature of appointment**		
Permanent	261	87%
Contractual	40	13%
**Qualification**		
Intermediate	258	86%
Graduation	35	11%
Master	8	3%
**Age (years)**		
22	27	9%
23	33	11%
24	108	36%
25	52	17%
26	30	10%
27	51	16%
**Experience (years)**		
1	18	6%
2	140	46%
3	74	25%
4	18	6%
5	51	17%

*All percentages are rounded up. *N* = 301.*

### Statistical Analysis

For understanding complex relationships, it is imperative to apply a more sophisticated multivariate data methodology for analysis (Hair et al., 2014). SmartPLS v. 3.2.7 was used to estimate measurement and structural models. Several reasons were to follow partial least squares structural equation modeling (PLS-SEM) approach. First, PLS-SEM is a substitute approach to the covariance-based SEM (CB-SEM), and it is used where a theory is under development, and fundamental purpose is focused on explaining the variance of outcome constructs (Hair et al., 2016). Second, PLS-SEM eliminates requirements regarding distributional assumptions because data analysis is based non-parametric techniques (Hair et al., 2016), and third, it can handle complex models relatively well (Vinzi et al., 2010).

## Results

Results of SEM have been reported under measurement and structural models (Hulland, 1999; Chin, 2010). A reflective measurement model was established, keeping in view the nature of hypothesized relationships and the nature of constructs. First, the measurement model was assessed on the basis of “reliability and validity” (Hair et al., 2016). The reliability of measurement/outer model is assessed through Cronbach’s alpha, rho-A, and composite reliability (CR), whereas validity has been evaluated through convergent validity [outer loadings and average variance extracted (AVE)] (Mela and Kopalle, 2002) and discriminant validity (cross-loadings and Fornell–Larcker criterion) (Lucas et al., 1996; Hair et al., 2016). All the alpha coefficients, CR estimates, values of rho-A, and AVE were above their cutoff values (Hair et al., 2013, 2016) except AVE of job-related gossips, which was 0.469. Second measure of reliability was assessed through CR (Bacon et al., 1995), and here, all the values were >0.60, hence approving the reliability of measurement model (Hulland, 1999).

For evaluating convergent validity, in the first attempt, items having outer loadings below 0.708 were checked against each variable. Indicators CNC2, CNC4, and CNC12 were deleted against cynicism due to low outer loading values; similarly, indicator WI6 pertaining to workplace incivility was excluded due to low outer loading. Some items such as CNC5, JRG2, JRG3, and WI8 were not dropped in spite of lower outer loading, i.e., <0.708, as AVE of respective constructs was within the acceptable range (Hair et al., 2016). AVE of job-related gossips was 0.469, which was less than the threshold value of 0.50. However, if the scale is newly developed and is in testing phase, then AVE values between 0.40 and 0.50 can be considered. Moreover, as noted by Malhotra et al., 2012, AVE is a strict measure of convergent validity, and convergent validity could be established on the basis of CR alone. Thus, lower AVE value for job-related gossips was considered in this study (Table 2).

**TABLE 2:** Indicator reliability, VIF, composite reliability, Cronbach’s alpha, average variance extracted.

**Construct**	**Indicator**	**Indicator reliability**	**VIF**	**Alpha**	**rho-A**	**CR**	**AVE**
Cynicism	CNC1	0.827	3.158	0.924	0.936	0.937	0.626
	CNC10	0.803	4.620				
	CNC11	0.756	2.236				
	CNC3	0.841	5.226				
	CNC5	0.635	1.805				
	CNC6	0.876	3.503				
	CNC7	0.708	1.809				
	CNC8	0.787	2.303				
	CNC9	0.859	4.037				
Job-related gossips	JRG1	0.782	1.319	0.73	0.787	0.813	0.469
	JRG2	0.628	2.056				
	JRG3	0.528	1.164				
	JRG4	0.755	1.443				
	JRG5	0.700	2.302				
Nonjob-related gossips	NJRG1	0.827	2.332	0.871	0.872	0.907	0.662
	NJRG2	0.841	2.316				
	NJRG3	0.789	1.830				
	NJRG4	0.722	1.453				
	NJRG5	0.880	3.009				
Psychological contract violation	PCV1	0.796	4.388	0.833	0.857	0.886	0.659
	PCV2	0.834	4.758				
	PCV3	0.803	1.786				
	PCV4	0.815	1.657				
Workplace incivility	WI1	0.705	4.228	0.862	0.875	0.894	0.547
	WI2	0.794	5.553				
	WI3	0.722	1.799				
	WI4	0.814	3.336				
	WI5	0.732	4.514				
	WI7	0.735	4.719				
	WI8	0.663	2.461				

*All factor loadings were significant at *p* < 0.01. VIF, variance inflation factor; CR, composite reliability; AVE, average variance extracted.*

For evaluating discriminant validity, cross-loadings and Fornell and Larcker (1981) criterion was assessed. Thus, it was established that square root of AVE of each latent construct was higher than the correlations among the latent constructs (Hair et al., 2011) (Table 3).

**TABLE 3:** Fornell and Larcker (1981) criterion.

**Variables**	**1**	**2**	**3**	**4**	**5**
1 Cynicism	**(0.791)**				
2 Job-related gossips	0.714	**(0.685)**			
3 Nonjob-related gossip	0.112	0.031	**(0.814)**		
4 Psychological contract violation	0.307	0.461	0.077	**(0.812)**	
5 Workplace incivility	0.342	0.513	0.062	0.411	**(0.739)**

**N* = 301. Values at the diagonal (bold and underlined) are square root of AVEs.*

### Assessment of Structural Model

To assess the structural model, we employed a bootstrapping procedure through 5,000 randomly drawn subsamples with replacement at 0.05% level of significance (Henseler et al., 2009; Hair et al., 2016). Assessment of the structural model has been tested through the coefficient of determination (level of *R*^2^) alternatively called predictive accuracy, effect size (*f*^2^), predictive relevance *Q*^2^, and path significance (Hair et al., 2013). Multicollinearity could decrease analytical impact of predicting construct (Mela and Kopalle, 2002); thus, to obtain the best parameter estimation assessment of multicollinearity is very necessary. According to Hair et al. (2013), variance inflation factor (VIF) must be <5. Here, almost all values were less than the cutoff value of +5.0, except indicators CNC3 and WI2 (Table 2). *R*^2^ represents combined effects of exogenous latent variables on endogenous latent variables. Here, workplace incivility showed 11% change in cynicism, and the combined effect of workplace incivility, cynicism, and PCV on job-related gossips was 62% showing a substantial effect (Henseler et al., 2009), whereas these entire constructs explained only 1% variation in nonjob-related gossips. Finally, workplace incivility explained 17% variation in PCV.

**FIGURE 1:**
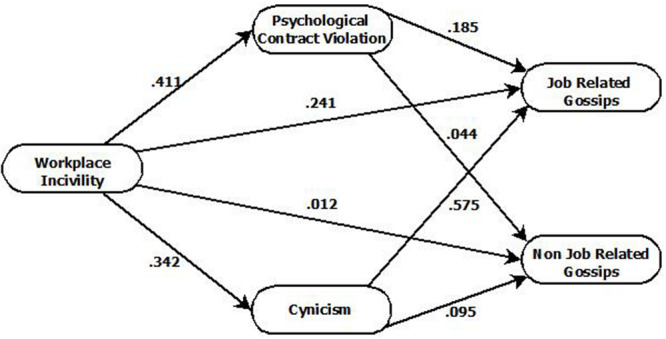
Path diagram.

Effect size (*f*^2^) is assessed as small, medium, and large (Cohen, 1992), and it is expressed as 0.02 (small), 0.15 (medium), and 0.35 and above (large). Here, very small size effect has been observed against nonjob-related gossips; the effect size predicting PCV due to workplace incivility was medium and small in the case of cynicism. Similarly, effect sizes against job-related gossips due to workplace incivility, cynicism, and PCV were observed as small, large, and small, respectively (Cohen, 1992). Predictive relevance was assessed through *Q*^2^ (Stone, 1974; Geisser, 1975). Values larger than 0 for a certain reflective endogenous latent variable indicate the path model’s predictive relevance, and in this study, values of *Q*^2^ were >0, implying that the model’s predictive relevance is correct (Chin, 1998). Finally, structural model is assessed on the basis of path estimation (direct, indirect, and total paths). Table 4 and Figure 1 presents the estimated value of path coefficients for direct, indirect, and total paths. Here, the path estimates between workplace incivility, cynicism, job-related gossips and PCV were significant at *p* < 0.05 (hypotheses H1, H2a, and H3a), whereas the path between workplace incivility and nonjob-related gossips (H2b) was insignificant at *p* < 0.05 (see Table 4). Similarly mediation was tested through variance accounted for (VAF) and newly synthesized approaches developed by Hair et al. (2016). No-effect non-mediation was observed in the case of H2d, and H3c as neither the direct effect nor the indirect effect was found significant for workplace incivility cynicism nonjob-related gossips as well as workplace incivility PCV nonjob-related gossips. On the other hand, complementary mediation was observed due to direct and indirect paths significance (Table 5) in the case of H2c (workplace incivility cynicism job-related gossips) and H3b (workplace incivility PCV job-related gossips).

**TABLE 4:** Hypotheses testing.

**Hypotheses**		**Beta**	**SD**	**T**	***p***	**Status**
**H:1**	Workplace incivility → cynicism	0.342	0.054	6.282	0.00	Supported
**H:2a**	Workplace incivility → job-related gossips	0.241	0.045	5.299	0.00	Supported
**H:2b**	Workplace incivility → nonjob-related gossips	0.012	0.068	0.169	0.87	Not supported
**H:3a**	Workplace incivility → psychological contract violation	0.411	0.045	9.069	0.00	Supported

**TABLE 5:** Hypotheses testing (mediation analysis).

**Hypotheses**		**Direct effect**		**Indirect effect**		**Total effect**	**VAF**	**Status**
		**Beta**	***p***	**Beta**	***p***			
**H:2c**	Workplace incivility → cynicism → job-related gossips	0.241	0.00	0.196	0.00	0.531	37%	Supported
**H:2d**	Workplace incivility → cynicism → nonjob-related gossips	0.012	0.87	0.032	0.18	0.062	52%	Not supported
**H:3b**	Workplace incivility → psychological contract violation → job-related gossips	0.241	0.00	0.076	0.00	0.531	14%	Supported
**H:3c**	Workplace incivility → psychological contract violation → nonjob-related gossips	0.012	0.87	0.032	0.51	0.062	52%	Not supported

### Discussion

First, the empirical findings of this study showed that lower-level employees tend to involve in gossips practice, which is related to their job when they experience incivility. These gossips may by regarding colleagues’ poor job performance, carelessness, poor work engagement, inexperience, and poor job knowledge, poor interpersonal skills, or lack of job morality (Cole and Dalton, 2009). Based on empirical grounds, it can be argued that gossip is considered an important communication device for expressing and managing emotions in organizations, as group members consider it as an important channel for sharing information and source to assure social bonding (Yang et al., 2014). These findings are also in line with the recommendations of Kuo et al. (2015) that gossip is a common phenomenon at work. Almost all employees are found to be engaged in hearing, making. or otherwise taking part in evaluative comments about other colleagues who are not present in the formal chit chat or conversation.

The findings of this study revealed an interesting situation regarding the relationship between workplace incivility and nonjob-related gossips. In this case, insignificant relationship was observed. There might be a reason for not involving in nonjob-related gossips such as discussing the sorrowful life events of colleagues, illness, poor interaction with children, divorce, separation, marital problems, or even poor relationships with family members. The reason for an insignificant relationship might be the prevalence of Islamic culture in Pakistan, an Islamic country where 99% of the population is Muslim, and in a Muslim society discussing the issues of someone behind him or her is considered unethical and against the preaching of Islam.

The positive relationship between workplace incivility and cynicism was also observed. It implies that employees experiencing workplace incivility tend to develop negative feelings regarding organizational policies, activities, goals, and performance. These findings are also in line with the argument of the COR theory. Furthermore, these findings also confirm that individuals experiencing incivility engage themselves in negative feelings when they end up using their personal resources (Schaufeli and Bakker, 2004; Bakker and Demerouti, 2007).

Similarly, a positive relationship among workplace incivility and PCV has been observed, which implied that individuals experiencing workplace incivility tend to develop perception that their employer has violated the unwritten agreement and they have been betrayed by their employer. The preposition of social exchange theory (Blau, 1964) that individuals tend to build links within organizational environment on the basis of exchange of socioeconomic benefits is confirmed. The complementary or partial mediation between workplace incivility and job-related gossips through cynicism showed that workplace incivility has small impact on job-related gossips through cynicism. The no-non-mediation situation for workplace incivility and nonjob-related gossips under cynicism and PCV (both mediating variables) provides important insights and needs further investigation in a non-Muslim nation’s context.

## Conclusion

Based on the empirical findings of this study, it can be concluded that workplace incivility develops feelings of cynicism among employees working at lower cadre. Furthermore, employees feel driven toward PCV when they experience incivility within the organizational circuits. This perception regarding PCV increases the tendency to involve in job-related gossips such as discussing the colleague’s poor job performance, carelessness, poor work engagement, inexperience and poor job knowledge, poor interpersonal skills, or lack of job morality. The relationship of workplace incivility in this study has been found much stronger with the other constructs of this study, i.e., nonjob-related gossips, cynicism, and PCV. The relationship of workplace incivility is very weak with nonjob-related gossips. Thus, it can also be concluded that individuals tend to involve in job-related gossips in spite of nonjob-related gossips while experiencing the incivility at workplace.

### Theoretical Contributions

From a theoretical perspective, this study has several contributions. First, this study has attempted to investigate the impact of workplace incivility in Asian society, which is characterized by personalized and identity-based interactions (Kakar and Kakar, 2007). Here, this study supported the existing literature that personalized and identity-based interactions due to kinship, caste, class, and religion may influence victims of workplace incivility who may suffer from negative outcomes such as cynicism, PCV, and job-related gossips (Ghosh, 2017). Second, perception of low-rank employees of a large public sector utility-based service organization was considered regarding workplace incivility by focusing on individuals of minority sociocultural status (LasisiOlukayode et al., 2014), which is a unique theoretical contribution of the study. This study confirmed that employees working at the low ranks of the organizational hierarchy in a service industry are less likely to be able to connect themselves with the management of the organization and hence engage in gossips (Lester et al., 2002). Third, this study also contributed to existing literature by exploring the mechanisms underlying the workplace incivility–gossips relationship. Furthermore, this study has attempted to answer the research calls raised by various researchers by exploring the relationship among PCV, job-related gossips, and nonjob-related gossips (Kuo et al., 2015) and investigating the uncivil behaviors from South Asian perspective (Ghosh, 2017). Finally, this set of variables was not tested before; thus, this study has tested a new set of variables in new settings by quantifying the impact of workplace incivility on PCV, organizational cynicism, job-related gossips, and nonjob-related gossips of low-ranked employees working in the public sector organizations of Pakistan.

The findings of this study supported the argument that individuals tend to involve in negative activities (try to cope) when the job demands are very high, and resources to meet these demands are low. It will create exhaustion among employees (Schaufeli and Bakker, 2004). Thus, this study endorsed the JD-R model in explaining the link between incivility and gossips (Bakker and Demerouti, 2007); it is also a theoretical contribution of the study. Furthermore, this study has also tested the phenomenon of social exchange theory (Blau, 1964) and endorsed that employees tend to build links within the organizational environment based on socioeconomic benefits. In addition to this, the present study also endorsed the affective events theory (Weiss and Cropanzano, 1996), which explains the phenomena of emotional reactions to the specific events that occur at the workplace. This study confirmed that individuals experiencing incivility show negative behaviors in the shape of negative job-related gossips, but relationship of incivility with nonjob-related gossips was found insignificant, which is in contradiction with the findings of previous researchers (Pate et al., 2003). Moreover, the results showed that PCV did not drive individuals to indulge in nonjob-related gossips; this is also a contribution of the study.

The present study has contributed an interesting finding into the literature that lower-level employees in Asian societies do not spread nonjob-related gossips such as discussing the sorrowful life events of colleagues, illness, and poor interaction with children, divorce, separation, marital problems, or even poor relationship with family members when they experienced incivility at the workplace. This finding is contradictory to the existing literature (Kuo et al., 2015).

### Practical Implications

The outcomes of this study portray that employees experiencing incivility at the workplace tend to spread gossips regarding negative aspects of the job such as colleague’s poor job performance and carelessness. Thus, it is obvious that individuals experiencing incivility will engage in activities that are non-productive for both, organization and individuals; thus, management should try to curtail the prevalence of the incivility in any form (bullying, aggression, abusive supervision) or from any source downward, upward, or lateral, within the organizational circuits in order to lessen the negative consequences. Furthermore, employees also tend to develop negative thinking regarding employers when they experience incivility so that it might reduce the positive image of the organization; thus, organizations should also formulate policies and procedures to eradicate workplace incivility. Technology can help organizations tackle incivility issues at the workplace, so use of electronic surveillance must be enhanced in order to control the incivility at workplace.

### Limitations and Future Research Directions

Last but not the least, this study also has some limitations, just like other cross-sectional investigations. First, data were collected under a survey method through a cross-sectional design, which does not confirm causality; thus, future research must follow longitudinal research design to confirm the causality. Furthermore, only lower-level employees were approached for data collection, which is a major limitation of this study; in the future, employees from other hierarchical levels must also be approached for data collection. Moreover, employees of a public sector organization were the population of this study where bureaucratic environment has a higher power distance, which might be a cause of incivility (Lammers and Stapel, 2009); thus, exploring the perceptions of the employees belonging to the private sector will bring important insights into the literature in the future. Future studies must focus on nonjob-related gossips as dependent variable in other settings. In the future, other dependent variables pertaining to coping strategies could be investigated along with these variables. Furthermore, employees’ resilience could also be examined in future studies. An additional constraint was the small size of the sample for this study. A large sample size might bring important results for theoretical and practical insights. Generalizability of these findings may be limited owing to the small size of the sample and a particular population. Thus, the generalizability of these results must be viewed only with great caution. Perhaps, a better picture would have been obtained if other sectors have been taken into consideration, as workplace incivility is a more common phenomenon that usually prevails in all organizations and across all cultures (Cortina et al., 2001).

In this study, incivility was investigated as a single-dimension construct, and no particular discrimination was made regarding downward, upward, or lateral incivility; future studies, therefore, could investigate other types of incivility, too. In addition to this, bystander experiences can also be taken under consideration in future studies for better picture. Gossip triad is a term used in the literature to denote gossip as a dynamic process, and its effects can be seen by the interaction of three components of gossip triad, i.e., gossiper, listener/respondent, and target (Michelson et al., 2010). Thus, in the future, gossips can be investigated as a triad.

Finally, this study focused on determining the effects of workplace incivility, PCV, cynicism, job-related gossips, and nonjob-related gossips in public sector organizations. Whereas this may be important for generalizability, it may also be limiting because power distribution companies might have a highly politicized environment due to presence of a strong worker’s union. Thus, it will be an important issue to investigate incivility in unionized as well as in non-unionized organizations, both in public and private sectors.

## Data Availability Statement

The datasets generated for this study are available on request to the corresponding author.

## Ethics Statement

The studies involving human participants were reviewed and approved by the Ethics Committee of Government College University Faisalabad, Pakistan. The participants provided their written informed consent to participate in this study.

## Author Contributions

MB conceptualized the research framework, while RS and SSal prepared the manuscript. MA provided technical support and leadership while SG collected the data. SSaq performed the data analysis and reported results and methodology.

## Conflict of Interest

SG was employed by the company Multan Electric Power Company (MEPCO).

The remaining authors declare that the research was conducted in the absence of any commercial or financial relationships that could be construed as a potential conflict of interest.
